# Urine Bisphenol-A Level in Relation to Obesity and Overweight in School-Age Children

**DOI:** 10.1371/journal.pone.0065399

**Published:** 2013-06-12

**Authors:** De-Kun Li, Maohua Miao, ZhiJun Zhou, Chunhua Wu, Huijing Shi, Xiaoqin Liu, Siqi Wang, Wei Yuan

**Affiliations:** 1 Division of Research, Kaiser Foundation Research Institute, Kaiser Permanente, Oakland, California, United States of America; 2 Department of Health Research and Policy, School of Medicine, Stanford University, Stanford, California, United States of America; 3 Department of Epidemiology and Social Science on Reproductive Health, Shanghai Institute of Planned Parenthood Research, & World Health Organization Collaborating Center for Research in Human Reproduction, Shanghai, China; 4 School of Public Health, Key Lab for Public Health Safety, & WHO Collaborating Center for Occupational Health, Fudan University, Shanghai, China; 5 Department of Child and Adolescent Health, School of Public Health, Fudan University, Shanghai, China; 6 National Population & Family Planning Key Laboratory of Contraceptive Drugs and Devices, Shanghai, China; NIDDK/NIH, United States of America

## Abstract

Bisphenol-A (BPA) is a potential endocrine disruptor impacting metabolic processes and increasing the risk of obesity. To determine whether urine BPA level is associated with overweight/obesity in school-age children, we examined 1,326 students in grades 4–12 from three schools (one elementary, one middle, and one high school) in Shanghai. More than 98% of eligible students participated. Total urine BPA concentration was measured and anthropometric measures were taken by trained research staff. Information on risk factors for childhood obesity was collected for potential confounders. Age- and gender-specific weight greater than 90^th^ percentile of the underlying population was the outcome measure. After adjustment for potential confounders, a higher urine BPA level (≥2 µg/L), at the level corresponding to the median urine BPA level in the U.S. population, was associated with more than two-fold increased risk of having weight >90^th^ percentile among girls aged 9–12 (adjusted odds ratio (aOR) = 2.32, 95% confidence interval: 1.15–4.65). The association showed a dose-response relationship with increasing urine BPA level associated with further increased risk of overweight (p = 0.006 for trend test). Other anthropometric measures of obesity showed similar results. The same association was not observed among boys. This gender difference of BPA effect was consistent with findings from experimental studies and previous epidemiological studies. Our study suggests that BPA could be a potential new environmental obesogen. Widespread exposure to BPA in the human population may also be contributing to the worldwide obesity epidemic.

## Introduction

In recent decades, both developing countries such as China as well as developed countries have witnessed an alarming increase in the prevalence of obesity [1]–[3]. The most troubling aspect of this increase is the acceleration in the prevalence of obesity and overweight among children. The prevalence of obesity in U.S. children is close to 20% [4]–[6]. This is especially alarming given the well-known consequences of overweight and obesity which include type 2 diabetes, hyperinsulinemia, insulin resistance, coronary heart disease, hypertension, stroke, and liver and kidney diseases among many other adverse health effects. While improving dietary habits and increasing physical activity have been the focus in reducing obesity, the rapid increase in the prevalence of obesity/overweight in countries with differing dietary styles and patterns of physical activity suggests the possible existence of other environmental risk factors. Emerging evidence linking the worldwide obesity epidemic to increased exposures to environmental endocrine disruptors, collectively called “environmental obesogens” [1], [6]–[9], has brought about an urgency to examine the role of exposure to these chemicals in relation to the obesity epidemic. One such important potential obesogen is bisphenol-A (BPA). Humans are widely exposed to BPA and animal studies have linked BPA to obesity [10]–[15].

Although it was first recognized in the 1930’s as a potential synthetic estrogen [16], BPA is contained in a variety of consumer products from baby bottles, plastic containers, and the resin lining of cans for food and beverages, to dental sealants [17]. Human populations are widely exposed to BPA in their daily lives. In a U.S. representative sample, BPA was detected in more than 92% of urine samples including those from children [17], [18] and in populations of many other countries [19]–[23]. One of the troubling aspects of BPA exposure is that younger children generally have higher urine BPA level than adults indicating a heavier biological burden likely due to a higher BPA exposure or slower metabolic process or both among young children [18].

In addition to affecting reproductive systems [23]–[27], an increased risk of obesity has emerged as an additional potential adverse effect of BPA exposure in experimental animal studies [1], [7], [9], [11]–[13]. Exposure to BPA has been shown to suppress the release of adiponectin [28]–[30], an adipocyte-specific hormone that increases insulin sensitivity. Thus, there is a biological plausibility that BPA could lead to insulin resistance and increased susceptibility to obesity and metabolic syndromes. BPA was shown to be a more effective adiponectin suppressor than estradiol, at a low environmental exposure level (0.1 nM) [28]. Given the widespread human exposure to BPA, a potential link between exposure to BPA and the risk of childhood obesity in human population has important public health implications.

Evidence of a relationship between BPA level and obesity in human populations is also emerging. Two studies have reported an association between urine BPA level and obesity in *adult* populations (>18 years of age) [31], [32]. One study reported an association between urine BPA level and metabolic diseases including diabetes [33]. In a recent study published in *JAMA*, Trasande et al reported an association between urine BPA and obesity among children and adolescents [34] using U.S. NHANES data. It should be noted NHANES was not specifically designed to examine BPA effect and consists of a largely non-Asian population. In 2011, we designed and conducted a population-based study to examine the relationship between urine BPA and obesity in school-age children. Given that animal studies have indicated that the BPA effect on obesity is observed only among females [11], we also examined the association for girls and boys separately.

## Methods

### Ethics Statement

The study was approved and overseen by the Institutional Review Boards of the School of Public Health, Fudan University, and Shanghai Institute of Planned Parenthood Research. The current study was an ancillary study to a larger national study of pubertal development and health of adolescents, which had already collected anthropometric measures and information related to pubertal development. The current study added some additional questions related to the risk factors for childhood obesity and collected additional urine samples. Parents of all students were sent a consent form which described the study purpose, the processes involved, including collection of urine samples, and voluntary nature of participation. Parents were asked to inform teachers if they did not want their children to participate in the study; but they were not required to sign the consent form due to infeasibility. All students were also informed by their teachers of the study purpose, process and voluntary nature of participation in advance, and reminded again at the time of data collection. This was the only process that was deemed feasible and accepted by the participating school district. The ethics committees/IRBs approved the opt out consent procedure for the students.

Schools in Jiading District, Shanghai, China were eligible for the study. Since the current study was an ancillary study, participants were not aware of the specific hypothesis of this study. In 2011, the three largest elementary, middle, and high schools of the district were selected, respectively, with all students from grades 4 through 12 being eligible for the study. Four classes of students from each grade were randomly selected so that about 150 students from each grade could be recruited (class size is typically about 40 students). Among the 1,451 eligible students, 18 students (1.2%) refused to participate.

### BPA Exposure

Spot urine samples (non-fasting) were collected from each participant. The collection time ranged from 9 am to 4 pm. All urine kits were made of BPA free materials. For each urine sample, the total urine BPA concentration (free plus conjugated species) was measured as in previous studies using HPLC (high performance liquid chromatography) [18] [35]–[37]. Urine samples were mixed with phosphate buffer and β-glucuronidase (Sigma Chemical Co., St. Louis, MO) for hydrolyzation. Afterwards, samples were extracted twice with ether: n-hexane (1∶1) (HPLC grade, Dikma) and supernatants were evaporated with nitrogen gas. The residue was dissolved in 40% acetonitrile and analyzed by HPLC with fluorescence detection (HPL/FD). The limit of detection (LOD) was 0.31 µg/L. This LOD level was comparable to that reported by previous studies [18]. The assay was conducted by co-authors at the Department of Occupational Health and Toxicology, School of Public Health & WHO Collaborating Center for Occupational Health, Fudan University, Shanghai, China. Detailed methodology involved in the assay has been published elsewhere [35]–[37]. We used BPA level of 2 µg/L as the cutoff (about the median urine BPA level in the U.S. population [17], [18]). Those with BPA level above the cutoff were compared to those with BPA level below the cutoff (high vs. low).

Seventy-two female students did not provide urine specimens because they happened to have menstrual periods on the day when the urine specimen was being collected, and urine samples of 17 students were accidentally damaged during transportation. Thus, 1,326 students were included in the final analyses (91.4% of the initial eligible population).

### Outcomes

Anthropometric measurements including weight, height, hip circumference, waist circumference, and skinfold thickness were taken by trained staff members at the time when urine BPA samples were collected. All methods of measurements were standardized. Calibrated instruments (e.g., scales and height measuring meters) were used. Given that BMI is not as reliable a measure of obesity in children as in adults and height in children is usually not as reliably measured as weight [4], we used weight as the primary measure of overweight/obesity. We used 90^th^ percentile age- and gender-specific population weight distribution as a cutoff for overweight based on the published survey (published information for 85^th^ percentile is not available) [38]. In addition, we used other parameters for measuring overweight/obesity including hip circumferences, waist circumference, waist to height ratio, skinfold thickness, and BMI as secondary measurements to verify the findings based on weight. We also used 90^th^ percentile of these parameters based on either the published standards, or if available, data from the study population [38].

### Potential Confounders

At the time of data collection, a food frequency questionnaire with 24 questions was administered to all participating students to determine their dietary patterns (e.g., frequency of eating junk food, unbalanced diet such as eating favorite foods only, and habit of eating fruits/vegetables). This questionnaire has been used in many previous studies with a similar Chinese population [39]. Information on physical activities (e.g., average daily time on playing video/computer games and participating in sports or other physical activities), parental overweight, and children’s current depression status using the published Children's Depression Inventory (CDI) [40] was also collected. Many of these factors are potential risk factors for childhood obesity which could be potential confounders if they were found to be related to urine BPA level. These factors were adjusted for or evaluated for possible interaction with the effect of BPA exposure on childhood obesity during analyses.

### Analysis

Univariate analyses were first conducted to examine the characteristics of the study population in relation to the urine BPA exposure level. For dichotomized variables, chi-square test was used to evaluate statistical significance. For continuous variables, t-test was used. Logistic regression with 95% confidence interval was used to estimate odds ratio of obesity measurements associated with urine BPA level after adjustment for potential confounders. Statistical significance was determined at α = 0.05. We used SAS 9.1 as the software for conducting analyses. Potential confounders, as described above, were adjusted for in all analyses evaluating the association between urine BPA level and obesity.

## Results

Table 1 presents the characteristics of participating students by their urine BPA levels. The factors in Table 1 include demographic variables (e.g., parental education) and potential risk factors for childhood obesity including dietary factors, physical activity, mental health (i.e., depression), and parental overweight. Overall, urine BPA level was not associated with the variables in Table 1 except that those students who spent more time playing video/computer games had a higher BPA level. Table 1 also showed that younger students had a slightly higher BPA level than older students. This finding is consistent with the findings from the U.S. population showing that BPA level is generally higher in younger children [18].

**Table 1:** Characteristics of Participating Students According to Urinary BPA Level**.**

		BPA level (µg/L)		p-value
		<2 (n = 748)	≥2 (n = 578)	
**Age**		13.50±2.68*	13.26±2.50*	p = 0.10
**Gender**	Male	372(55.44%)	299(44.56%)	
	Female	376(57.40%)	279(42.60%)	p = 0.47
**School**	Elementary	185 (54.41%)	155 (45.59%)	
	Middle school	330 (55.56%)	264 (44.44%)	
	High school	233 (59.44%)	159 (40.56%)	p = 0.33
**Residence**	Urban	570 (56.77%)	434 (43.23%)	
	Rural	113 (54.85%)	93 (45.15%)	p = 0.67
**Paternal Education**	High school or less	490 (57.38%)	364 (42.62%)	
	College & above	212 (54.22%)	179 (45.78%)	p = 0.33
**Maternal Education**	High school or less	496 (57.41%)	368(42.59%)	
	College & above	209 (54.86%)	172 (45.14%)	p = 0.44
**Paternal overweight**	Yes	253 (57.24%)	189 (42.76%)	
	No	461 (56.43%)	356 (43.57%)	p = 0.82
**Maternal overweight**	Yes	119 (56.40%)	92 (43.60%)	
	No	591 (56.61%)	453 (43.39%)	p = 1.00
**Playing video/computer games**	≥30 min/day	327 (53.17%)	288 (46.83%)	
	<30 min/day	421 (59.21%)	290 (40.79%)	p = 0.03
**Unbalanced diet** ***	Yes	302 (57.41%)	224 (42.59%)	
	No	407 (57.49%)	301 (42.51%)	p = 1.00
**Eating Junk foods**	Regularly (>5 days/week)	201 (57.59%)	148 (42.41%)	
	Not regularly	547 (55.99%)	430 (44.01%)	p = 0.65
**Eating vegetables**	Regularly (every day)	490 (56.98%)	370 (43.02%)	
	Not regularly	258 (55.36%)	208 (44.64%)	p = 0.61
**Eating fruits**	Regularly	500 (57.01%)	377 (42.99%)	
	Not regularly	248 (55.23%)	201 (44.77%)	p = 0.58
**Depression scores** **	≥median (>10)	366 (56.92%)	277 (43.08%)	
	<median	382 (55.93%)	301 (44.07%)	p = 0.75
**Sports activities**	≥30 min/day	309 (55.88%)	244 (44.12%)	
	<30 min/day	439 (56.79%)	334 (43.21%)	P = 0.78

The numbers in individual categories may not match the total numbers due to missing values.

* Mean and SD.

** Children's Depression Inventory (CDI)^34^.

*** Eating only favorite foods.

Table 2 shows the relationship between urine BPA level and overweight in female and male students separately. High urine BPA level was associated with overweight among female students, particularly among girls entering the pubertal stage of development (9–12 years old): those with urine BPA level at 2 µg/L or higher (above the median in this population) had more than twice the risk of being overweight (>90^th^ percentile of age- and gender-specific population weight distribution) than those whose urine BPA level <2 µg/L (adjusted odds ratio was 2.32, 95% confidence interval: 1.15–4.65) (Table 2). Urine BPA level was not associated with overweight/obesity for older female students (>12 years of old) (Table 2).

**Table 2:** Urine Bisphenol-A (BPA) Level in Relation to Overweight among School-age Children.

Age	BPA level	Weight >90th		Crude OR	Adjusted OR*
	µg/L	No	Yes	(95% CI)	(95% CI)
***Among Girls***					
All	<2	310 (82.45%)	66 (17.55%)	Reference	Reference
	≥2	220 (78.85%)	59 (21.15%)	1.26	1.29
				(0.85–1.86)	(0.83–2.01)
9–12	<2	114 (78.62%)	31 (21.38%)	Reference	Reference
	≥2	62 (63.92%)	35 (36.08%)	**2.08**	**2.32**
				(1.17–3.68)	(1.15–4.65)
>12	<2	196 (84.85%)	35 (15.15%)	Reference	Reference
	≥2	158 (86.81%)	24 (13.19%)	0.85	0.90
				(0.49–1.49)	(0.48–1.72)
***Among Boys***					
All	<2	281 (75.54%)	91 (24.46%)	Reference	Reference
	≥2	229 (76.59%)	70 (23.41%)	0.94	0.82
				(0.66–1.35)	(0.55–1.23)
9–12	<2	84 (71.19%)	34 (28.81%)	Reference	Reference
	≥2	83 (72.81%)	31 (27.19%)	0.92	0.71
				(0.52–1.64)	(0.34–1.45)
>12	<2	197 (77.56%)	57 (22.44%)	Reference	Reference
	≥2	146 (78.92%)	39 (21.08%)	0.92	0.87
				(0.58–1.46)	(0.52–1.45)

* **Odds ratio** adjusted for those factors listed in Table 1 (age, gender, school, residence, paternal and maternal education and overweight, playing video games, unbalanced diet, eating junk food, vegetables or fruit, depression scores and sports/activities).

Urine BPA level was not associated with overweight/obesity among male students (Table 2). The interaction term for the association between genders was borderline significant (p = 0.05).

To further examine the association between urine BPA level and overweight among girls aged 9–12 years old, we examined the dose-response relationship. Increasing urine BPA level was associated with increased risk of overweight in this group of girls. Compared to lower urine BPA levels (<50^th^ percentile), the risk of overweight was almost doubled for girls with urine BPA levels in the 50^th^ to 90^th^ percentiles, and further increased by more than 5 fold among girls whose urine BPA level was above the 90^th^ percentile (Table 3). The trend test for the dose-response relationship was highly significant (p = 0.006).

**Table 3:** Dose-response of Urine Bisphenol-A (BPA) Level in Relation to Overweight among School-age Girls 9 to 12 Years Old.

BPA level Percentile	Weight >90^th^		Adjusted Odds Ratio*
Percentile (µg/L)	No	Yes	(95% CI)
<50^th^ (0.98)	95 (79.17%)	25 (20.83%)	
50^th^–75^th^ (0.98–4.13)	43 (70.49%)	18 (29.51%)	1.92
			(0.79–4.66)
75^th^–90^th^ (4.13–10.04)	24 (64.86%)	13 (35.41)	2.04
			(0.77–5.41)
>90^th^ (10.04)	14 (58.33%)	10 (41.67%)	**5.18**
			(1.68–15.91)

•Trend test: p = 0.006.

* Adjusted for those factors listed in Table 1 (age, gender, school, residence, paternal and maternal education and overweight, playing video games, unbalanced diet, eating junk food, vegetables or fruit, depression scores and sports/activities).

To examine whether urine BPA level is also associated with other measurements of overweight/obesity in this age group of girls, we examined urine BPA level in relation to hip circumference, waist circumference, waist- to-height ratio, skinfold thickness, and BMI. A high urine BPA level was consistently associated with greater risk of overweight measured by all of these parameters. The odds ratios were in the range of 1.47 to 2.88 although only the association with hip circumference reached statistical significance while the association with waist circumference was borderline significant (Table 4).

**Table 4:** Urine Bisphenol-A (BPA) Level in Relation to Other Measurements of Obesity among School-age Girls 9 to 12 Years Old.

Obesity measurement		BPA level	
		<2	≥2
**Hip circumference**	<90th	134 (92.41%)	82 (84.54%)
	≥90th	11 (7.59%)	15 (15.46%)
***Adjusted odds ratio*** *		Reference	**2.88**
(95% CI)			(1.12–7.45)
**Waist circumference**	<90th	134 (92.41%)	83 (85.57%)
	≥90th	11 (7.59%)	14 (14.43%)
***Adjusted odds ratio*** *		Reference	**2.60**
(95% CI)			(0.98–6.91)
**Waist-height ratio**	<90th	135 (93.10%)	84 (86.60%)
	≥90th	10 (6.90%)	13 (13.40%)
***Adjusted odds ratio*** *		Reference	**2.38**
(95% CI)			(0.92–6.16)
**Skinfold thickness**	<90th	132 (91.03%)	84 (86.60%)
	≥90th	13 (8.97%)	13 (13.40%)
***Adjusted odds ratio*** *		Reference	**1.86**
(95% CI)			(0.73–4.71)
**BMI**	<90th	118 (81.38%)	72 (74.23%)
	≥90th	27 (18.62%)	25 (25.77%)
***Adjusted odds ratio*** *		Reference	**1.47**
(95% CI)			(0.71–3.05)

* **Adjusted for those factors listed in** **Table 1** (age, gender, school, residence, paternal and maternal education and overweight, playing video games, unbalanced diet, eating junk food, vegetables or fruit, depression scores and sports/activities).

## Discussion

In this population-based epidemiological study of young school-age children, we observed that high urine BPA level was associated with overweight among female students aged 9–12 years old (likely in pubertal developmental stages) [41], but not in male students. This finding is consistent with findings in experimental animal studies where exposure to high BPA level led to weight gain in females, but not in males [10], [12], [42]. Human studies have also reported a gender difference in the BPA effect on other outcomes [41]. More generally, it has been shown that environmental risk factors more likely impact girls’ than boys’ weight [43]. Results from this study are also consistent with a recently published report showing that urine BPA was associated with obesity in children and adolescents [34]. Although the investigators did not observe a gender difference in the association, they reported an ethnic difference with an absence of the association in Hispanic children [34].

Our finding of the association of urine BPA level and overweight among girls aged 9–12 is consistent with findings from experimental studies [42]. BPA is an environmental estrogen which could both accelerate girls’ pubertal development and weight gain during this period. The acceleration of growth by BPA may impact both weight and height, leading to a slightly weaker BPA effect on BMI measurement. A lack of the association among older girls is supported by the observation of a compensatory delay in the post-pubertal period among the girls who had earlier accelerated maturation [44]–[47].

The consistency between our observation and previous experimental studies as well as other human studies provides support for a genuine underlying association. The cross-section nature of the study design is a limitation of the study. However, it is unlikely that our findings were due to overweight leading to a higher urine BPA level in girls entering the pubertal stage for the following reasons. First, we did not observe the same association among older girls (>12 years old). Had overweight led to higher urine BPA levels, one would have expected to observe the same relationship in older girls. Second, we did not observe a similar association among boys either. Again, this gender discrepancy of the association cannot be explained by obesity as a cause for high urine BPA. On the other hand, this gender difference can be explained by the findings from experimental studies showing gender-specific BPA effect on obesity, and other human studies showing gender difference of environmental risk factors for body weight [10]–[12]. Both dose-response relationship and gender-specific effect of BPA observed in our study are consistent with the findings from experimental studies [11]. Such consistency makes it less likely that the findings be a result of other factors.

There are multiple mechanisms by which BPA could lead to obesity through its adverse impact on the metabolic process. BPA has been shown to act on adipocytes and suppresses the release of adiponectin in human adipose tissues which could lead to insulin resistance and metabolic syndrome [11], [28]. BPA, like DES, acts on estrogen receptors which could lead to obesity in a gender-specific and dose-response manner [1], [7], [14], [48]. This may explain the observed gender-specific effect. Other mechanisms include BPA effect on the pancreas, thyroid hormone pathways and brain functions [10], [11], [31], [43].

We have controlled for many known risk factors for childhood obesity including dietary factors and physical activity. In our study population, urine BPA did not seem to be related to many risk factors for obesity. Another potential limitation is that we did not have information on potential confounders during pregnancy including maternal gestational diabetes, birthweight, or preterm delivery. Also, our sample size was not large enough for subgroup analysis which might have led to the inability to reach statistical significance for some estimates. Finally, only one spot urine sample was collected in our study. However, urine BPA has been reported to be relatively stable [49], [50]. It has also been shown that a single spot urine sample provides a reasonably good measure of true BPA exposure level once the sample size is reasonably large [51]. Any variation in urine BPA would have resulted in non-differential misclassification of BPA exposure level that is independent of outcome. Existence of such misclassification would have resulted in under-estimation of the strength of the observed association.

The strengths of the study include a high participation rate, multiple pieces of supporting evidence including dose-response relationship and consistent results using other obesity measures (though some did not reach statistical significance), and being consistent with findings from experimental animal studies and a recent human study [34]. The previously reported human study was based on NHANES data from the U. S. population. Our finding was based on a Chinese population. A similar association observed in different populations increases the likelihood for a true underlying association.

Evidence is accumulating that obesogens such as BPA exposure may be contributing to the worldwide obesity epidemic. As a potential environmental obesogen, BPA exposure warrants particularly careful examination given the widespread human exposure, especially considering that the exposure level is higher in young children [18]. Both of these facts (an obesogen to which a vast majority of the population are being exposed, and higher exposure level in children) could have important implications for the epidemic of childhood obesity.

### Conclusions

Our study indicated that exposure to high BPA level may contribute to childhood obesity. However, prospectively conducted studies with a clear time sequence between BPA exposure and obesity measurement, including long-term follow-up should be carried out to examine this important relationship.
